# Supplementary material for: Pathogenic Carboxyl Ester Lipase (CEL) Variants Interact with the Normal CEL Protein in Pancreatic Cells
Source: Cells. 2020 Jan 18;9(1):244. doi: 10.3390/cells9010244 (PMC7017060; doi:10.3390/cells9010244)
Supplement: Supplementary file 1 [file cells-09-00244-s001.pdf]

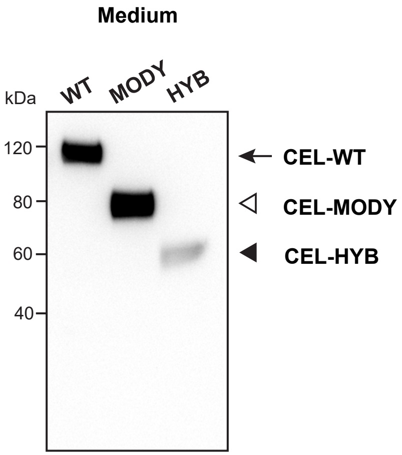

Supplementary Figure 1. Representative western blot showing varying secretion of CEL protein variants into the medium. HEK293 cells stably expressing CEL variants were grown for 48 h towards 80-90 % confluence. The CEL-MODY protein usually appeared more abundant in the medium than CEL-WT (see also reference [21]), whereas CEL-HYB was less well secreted. When studying CEL reuptake, we therefore used undiluted conditioned medium for CEL-HYB. For CEL-WT and CEL-MODY, the conditioned media were diluted in DMEM to 50% and 33%, respectively.
